# Mechanistic studies of tumor-associated macrophage immunotherapy

**DOI:** 10.3389/fimmu.2024.1476565

**Published:** 2024-09-30

**Authors:** Jiandong Cao, Chengsen Liu

**Affiliations:** ^1^ Department of Thoracic Surgery, Shenyang Chest Hospital & Tenth People’s Hospital, Shenyang, Liaoning, China; ^2^ Department of Radiotherapy, The People’s Hospital of Liaoning Province, Shenyang, Liaoning, China

**Keywords:** tumor-associated macrophages, TAMs, tumor microenvironment, immunotherapy, cancer

## Abstract

Tumor-associated macrophages (TAMs) are present in the tumor microenvironment and can polarize into subtypes with different functions and characteristics in response to different stimuli, classifying them into anti-tumorigenic M1-type and pro-tumorigenic M2-type. The M1-type macrophages inhibit tumor growth through the release of pro-inflammatory cytokines, whereas the M2-type macrophages contribute to tumor progression through the promotion of tumor proliferation, angiogenesis and metastasis. Due to the duality of macrophage effects on tumors, TAMs have been a hot topic in tumor research. In this paper, the heterogeneity and plasticity of TAMs, the interactions between TAMs and other immune cells, and the effects of TAMs on tumors are reviewed, and the therapeutic strategies for TAMs are summarized and discussed. These therapeutic strategies encompass methods and approaches to inhibit the recruitment of TAMs, deplete TAMs, and modulate the polarization of TAMs. These studies help to deeply understand the mechanism of TAMs-tumor interaction and provide reference for combination therapy of tumors.

## 1 Introduction

The innate and adaptive immune systems in the human body are able to recognize and eliminate tumors (1, 2), but tumors may still be able to escape from the immune system and establish an immunosuppressive tumor microenvironment (TME) that is conducive to tumor progression through the modulation of immune cell function (3, 4). Macrophages are an important component of the innate immune system and are highly plastic and heterogeneous. Macrophages are polarized into classically activated M1-type and alternatively activated M2-type under different environmental conditions (5, 6). M1-type macrophages, as a potent anti-tumor immune cell, express high levels of markers (human: CD68, CD80, CD86, MHC-II, IL-1R, IL-12, TLR-2, TLR-4 and inducible nitric oxide synthase 2 (iNOS2; mice: CD68, CD80, CD86, MHC-II, IL-12, IL-23), and secrete a variety of inflammatory cytokines, such as interleukin-1β (IL-1β), IL-6, and IL-12, to exert anti-inflammatory and tumor-suppressive effects (7). In contrast, M2-type macrophages express different markers and perform distinct roles in humans and mice. In humans, M2-type macrophages express markers such as CD86, CD163, CD206, CD200R, CD209, CD301, IL-1R, IL-10, TLR-1, TLR-8, and VEGF. In mice, they express markers like arginase-1, found in the inflammatory zone 1 (FIZZ1), and Ym1/2. M2-type macrophages are recruited by tumor cells into the TME to promote tumor progression (8). Within the TME, these macrophages are referred to as tumor-associated macrophages (TAMs), which actively produce cytokines that promote angiogenesis and support tumor cell survival and metastasis. In addition, TAMs express immunosuppressive factors, such as IL-10 and transforming growth factor-β (TGF-β), which play a crucial role in suppressing anti-tumor immune responses (9). In addition, depletion of TAMs (10) or conversion of macrophages to anti-tumor M1-type (11) significantly reduces tumor cell growth. Targeting TAMs in TME has evolved as an effective cancer immunotherapy strategy. This strategy combines traditional or emerging immunotherapies for synergistic effects and has important applications in cancer treatment.

**Figure 1:**
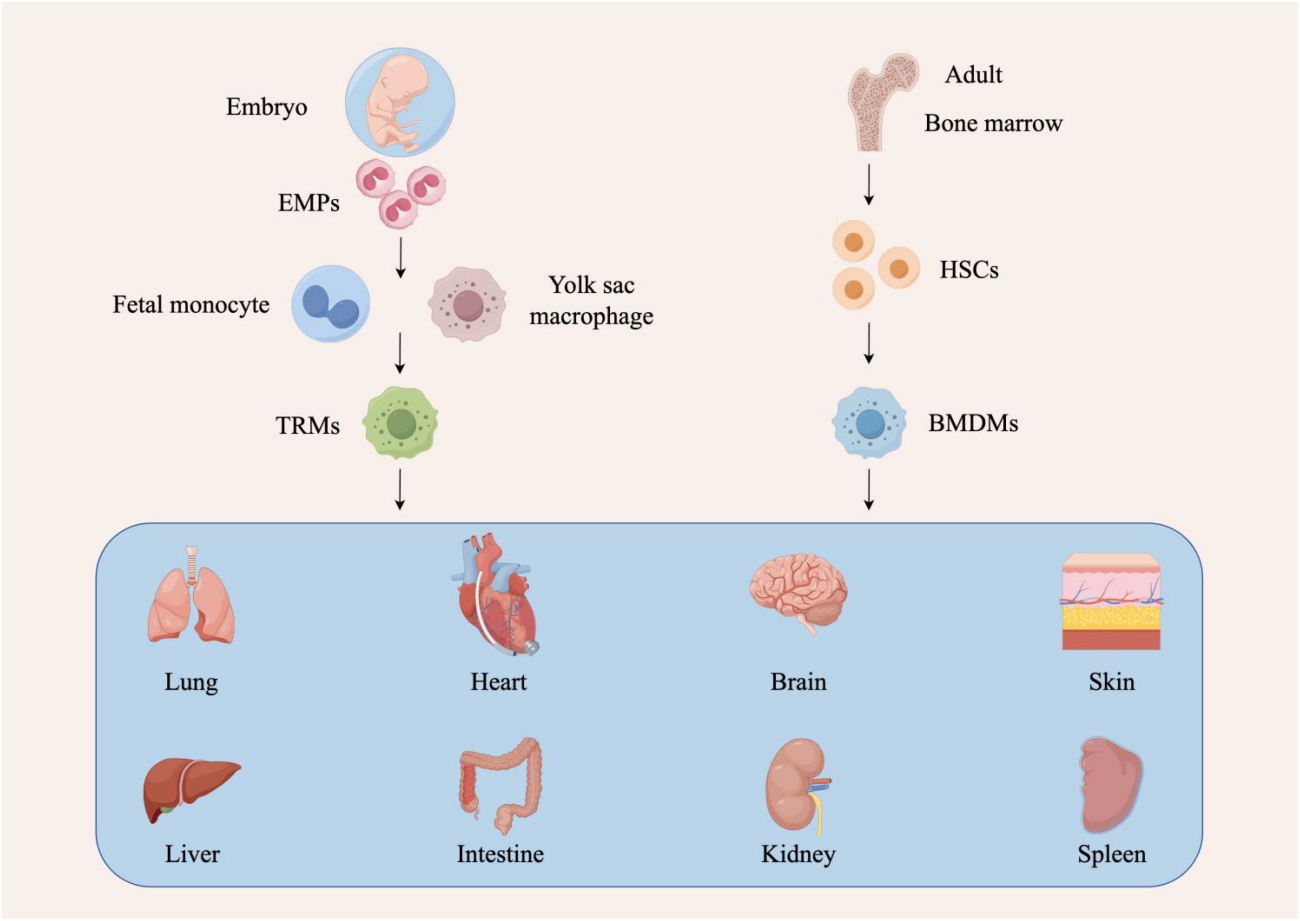
Different sources of tumor-associated macrophages. There are two sources of macrophages. The first source originates from hematopoietic stem cells (HSCs) in the bone marrow. These cells undergo developmental and differentiation steps, enter the peripheral blood as bone marrow-derived macrophages (BMDMs), and migrate to different tissues in response to stimuli. Depending on the tissue they enter, these macrophages are given different names, such as Kupffer cells in the liver, alveolar macrophages in the lungs, and microglia in the central nervous system. The second source is of embryonic origin, deriving from erythro-myeloid progenitors (EMPs) in the yolk sac and fetal liver, which develop into tissue-resident macrophages (TRMs).

Macrophages originate from the embryonic yolk sac, fetal liver, and bone marrow, and are categorized into two types: bone marrow-derived macrophages (BMDMs) and tissue-resident macrophages (TRMs) (12, 13). BMDMs are derived from hematopoietic stem cells in the bone marrow, while TRMs are generated from erythro-myeloid progenitors (EMPs) in the yolk sac and fetal liver (14). Macrophages from different sources within the same tissue can have distinct roles. For instance, in lung, brain, and pancreatic tumors, TAMs derived from hematopoietic stem cells are more likely to express genes associated with immunosuppression and antigen presentation, whereas embryonically-derived TAMs express genes linked to tissue remodeling and wound healing (15, 16). The heterogeneity and plasticity of TAMs, influenced by their different origins, contribute significantly to the complexity of the tumor microenvironment (TME) (**Figure 1**).

**Figure 2:**
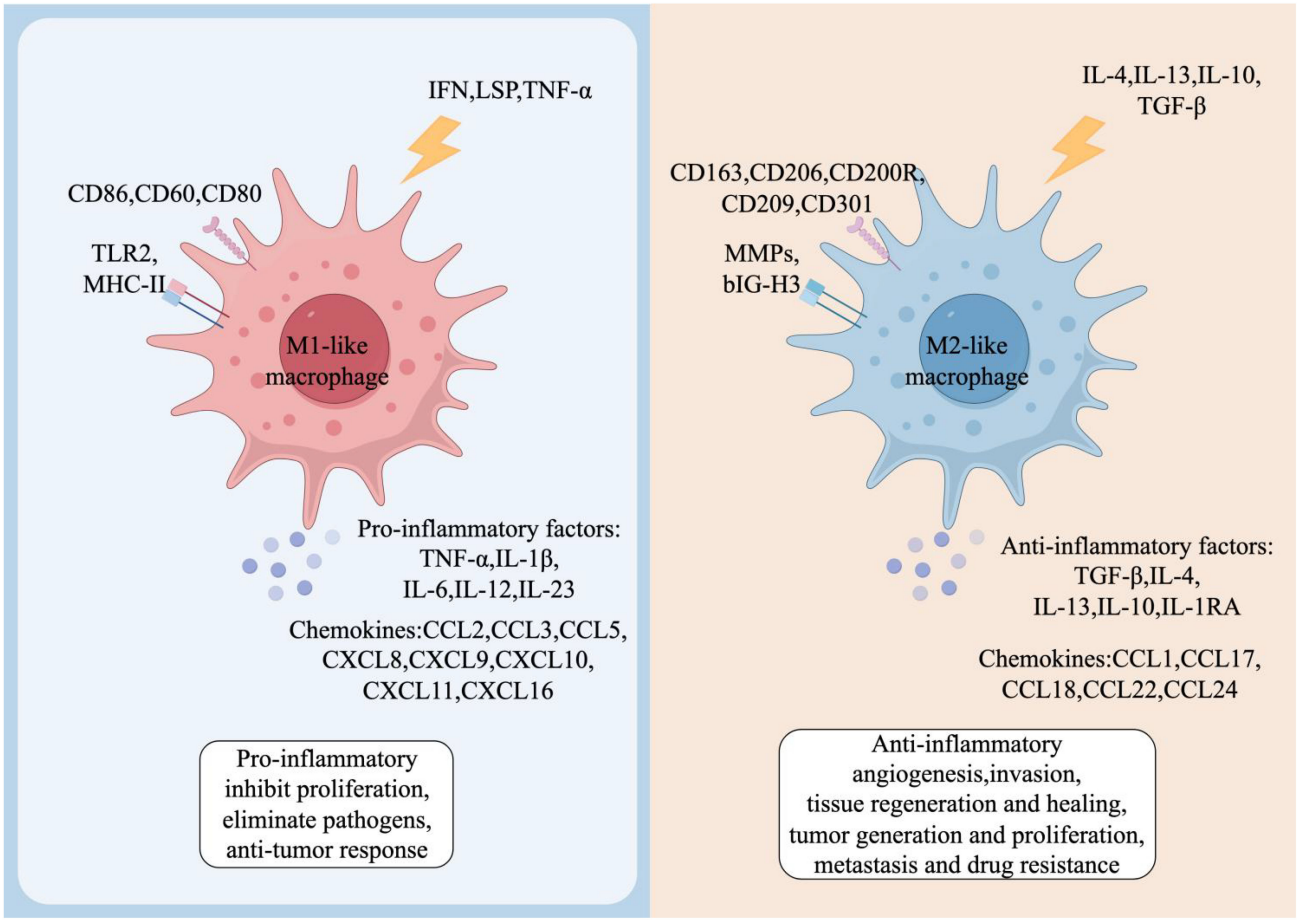
Phenotypes and functions of TAMs. Both M1-like and M2-like TAMs have distinct cell surface markers and functional factors. M1-like TAMs are induced by interferon-γ (IFN-γ), lipopolysaccharides (LPS) and tumor necrosis factor-α (TNF-α). These macrophages exhibit a pro-inflammatory phenotype and produce cytokines including TNF-α, interleukin-1β (IL-1β), and IL-6, among others. In the TME, M1-like TAMs promote inflammation, inhibit proliferation, eliminate pathogens, and contribute to anti-tumor responses. Conversely, M2-like TAMs are induced by IL-4, IL-13, IL-10 or transforming growth factor-β (TGF-β), and produce anti-inflammatory cytokines, such as IL-10 and TGF-β. M2-like TAMs in the TME are involved in anti-inflammatory activities, promoting angiogenesis, influencing tissue regeneration and healing, and fostering tumor growth, proliferation, metastasis, and drug resistance.

Activated macrophages can either kill tumor cells and induce antitumor activity or promote tumor growth and metastasis (17, 18). Further studies revealed that this duality is due to differences in macrophage stimulatory factors and secreted products resulting in both M1 and M2 phenotypes of macrophages in malignant tumors (19). Stimulated by pro-inflammatory factors such as interferon (IFN)-γ, lipopolysaccharide (LPS), and tumor necrosis factor (TNF)-α, macrophages exhibit the M1 phenotype, which is capable of generating inflammatory responses, exerting anti-tumor effects, and promoting anti-tumor immune responses through the release of IL-1β, IL-12, and reactive oxygen/nitrogen intermediates (20). In contrast, macrophages induced in TME can also exhibit M2-type characteristics. Induced by anti-inflammatory stimuli such as IL-4, IL-10, IL-13, glucocorticoids and immune complexes, macrophages secrete high levels of IL-10 and increase the expression of mannose receptors and galactose receptors (21), thus acting as an anti-inflammatory agent to promote wound healing and tissue repair, as well as to promote proliferation, metastasis, angiogenesis, and endocytosis of tumor cells (**Figure 2**).

A proper balance between M1 and M2-type macrophages is essential for maintaining tissue homeostasis (22). However, a large body of evidence suggests that the widely used ratio of M1/M2 macrophages does not accurately reflect the inflammatory state of tissues because of the stimulation of multiple pro- and anti-inflammatory factors in the tissue microenvironment. Influenced by these stimuli, macrophages do not have a defined direction of polarization when recruited to specific tissues, but rather exhibit a high degree of dynamism and heterogeneity. Thus, a synthesis of the various stimulus signals is likely to be more conducive to a comprehensive and in-depth understanding of the activated subpopulations of macrophages. Some of the more important of these signals include individual occurrence-related signals, tissue-specific signals, and other exogenous/endogenous signals (23).

## 2 Major molecules regulating TAM function

TAM immunoregulatory mechanisms include the colony stimulating factor 1 (CSF-1)/colony stimulating factor 1 receptor (CSF-1R) axis, IL-4/IL-13 and JAK/STAT6 transduction pathways, Toll-like receptor (TLR), and CD47-SIRPα signaling pathway (24). The CSF-1/CSF-1R axis affects tumor growth and metastasis by activating the phosphatidylinositol-3-hydroxy kinase (PI3K) signaling cascade and regulating the M1/M2 polarization of macrophages; IL-4/IL-13 and the JAK/STAT6 pathway are involved in the Th2-type immune response, inducing TAMs toward M2 phenotype and promoting abnormal tumor angiogenesis and progression; TLRs affect lung cancer metastasis and growth by recognizing pathogen-associated molecules and subsequently altering macrophage activation status; the CD47-SIRPα signaling pathway promotes tumor growth and metastasis by inhibiting macrophage-mediated phagocytosis. The study of these immunoregulatory mechanisms provides new ideas and targets for tumor therapy.

### 2.1 CSF-1/CSF-1R

Granulocyte-macrophage colony stimulating factor (GMCSF) regulates hematopoietic cell production and differentiation, and also plays a role in angiogenesis (25). CSF-1 binds to CSF-1R, which further promotes protein kinase B and mammalian target of rapamycin 2 (mTORC2) through activation of the PI3K signaling cascade, further promoting the activation of protein kinase B and mTORC2, thereby regulating the M1/M2 polarization axis in macrophages (26). Activation of PI3K and AKT kinases or overexpression can inhibit M1-type macrophage activation, and activation of the PI3K pathway mediates negative regulation of the nuclear factor-κB (NF-κB) signaling pathway that can promote M1 production (27). Additionally, CSF1R can be activated by binding to IL-34 (28). Therefore, when IL-34 is highly expressed with CSF-1R in tumors it marks tumor progression and lower survival. A study (29) demonstrated that high expression of IL-34 and M-CSF and their ligands was associated with lower survival in a cohort of lung cancer patients, because lung cancers with high IL-34 and M-CSF expression were more likely to progress to advanced stages. In addition, CSF-1 can produce factors that promote tumor growth and metastasis by recruiting and reprogramming TAM (30).

### 2.2 IL-4/IL-13 and JAK-STAT6

IL-4 and IL-13, which are involved in Th2-type immune responses (31), are among the major stimuli that induce TAM tendency toward the M2 phenotype that promotes abnormal tumor angiogenesis and tumor progression. IL-13 and IL-4 promote the phosphorylation of JAK by binding to type I IL-4 receptor (IL-4Rα and IL-4Rγc) and type II IL-4 receptor (IL-4Rα and IL-13Rα1), which in turn phosphorylates the transcription factor STAT6. Subsequently, activated STAT6 dimerizes and translocates into the nucleus, where it binds to the corresponding site of DNA, initiating the transcription of the target gene (32, 33). STAT6 activation also promotes the expression and transcription of M2-associated specific genes, such as Arg-1, Mrc-1, and Chil3/Ym1 (34). STAT6 acts as a key factor in IL-4 and IL-13 mediated macrophage polarization towards an immunosuppressive phenotype, and is also regulated by other factors. For example, one study (35) found that TRAF3 promotes STAT6 ubiquitination and transcriptional activity as shown by ubiquitination assay and luciferase assay. Site mutation analysis revealed that ubiquitination at STAT6 K450 plays a crucial role in TRAF3-mediated STAT6 activation, which promotes increased expression of M2-associated surface markers as well as tumor progression. Bone marrow TRAF3 deficiency was found to inhibit tumor growth and lung metastasis *in vivo* using a B16 melanoma mouse model.

### 2.3 TLRs

The body’s immune response to the environment can be divided into two types: innate immunity and adaptive immunity, and pattern recognition receptors (PRRs) are essential for the functioning of innate immunity (36, 37). In the tumor microenvironment, the interaction between pathogen-associated molecular patterns (PAMPs) and PRRs, especially TLRs, play a crucial role in tumor initiation and progression. TLRs can recognize different types of PAMPs, such as bacterial lipopolysaccharides and viral RNA. Although these PAMPs typically originate from infectious pathogens, in the tumor microenvironment, tumor cells or surrounding immune cells may also activate TLRs by releasing PAMP-like substances (38).

To date, a total of 13 TLRs have been identified in mammals, of which 11 are expressed in humans (TLR1-10). Macrophages can be reprogrammed through the activation of different TLRs, thereby altering the activation state of macrophages. For example, in a lung metastasis model, TLR4 can promote the effect of TAM on lung tumor metastasis through the NF-κB pathway. By using TLR4-deficient mice, it was found that TAM lacking TLR4 could not produce pro-inflammatory cytokines, nor angiogenic factors, and failed to activate NF-κB activity in tumors, thereby failing to inhibit (39). In addition, the Lewis lung carcinoma (LLC) cell line is a potent activator of macrophages. LLC-conditioned medium activates TLR2 and TLR6 through the extracellular matrix proteoglycan versican, leading to the production of TNF-α and IL-6 by macrophages, which strongly promotes lung cancer metastasis and growth (40). It has also been shown (41) that up to a 100-fold increase in M1-type macrophage production can be achieved by applying less toxic IFNs (including IFN-α and IFN-β) in combination with TLR agonists. This fully demonstrates their potential for anti-tumor development and suggests a new approach to TLR-related tumor immunotherapy.

### 2.4 CD47-SIRPα

Integrin-associated protein (IAP or CD47) is a receptor for members of the platelet-responsive protein family that regulates a range of cellular activities, including platelet activation, cell motility and adhesion, and leukocyte adhesion, migration, and phagocytosis (42). CD47 is an immunoglobulin widely distributed on the cell surface that inhibits phagocytosis of tumors by macrophages in order to promote growth and metastasis, and can be involved in the mediation of cell proliferation, migration, apoptosis and immune homeostasis (43). SIRPα, a transmembrane protein highly expressed on cell membranes, is the main ligand for the CD47 molecule (44). The NH2 terminus of its extracellular domain can bind to CD47, leading to tyrosine phosphorylation on the immunoreceptor tyrosine-based inhibitory motif (ITIM). This binding triggers the release of an inhibitory phagocytosis signal, which can inhibit macrophage-mediated phagocytosis, thereby protecting normal cells from damage caused by the immune system (45). CD47 has been shown to be highly expressed in a variety of solid tumors and correlates with a poor prognosis of tumors; therefore, inhibition of the CD47-SIRPα pathway enhances the body’s adaptive immune response and increases macrophage phagocytosis.

## 3 Interactions between TAMs and other immune cells

Crosstalk between TAMs and other immune cells is an important aspect of TAMs affecting tumor immunity. In addition to macrophages, TMEs contain several immune cell populations such as T-cells, B-cells, natural killer (NK) cells, and neutrophils, which interact with each other through different signaling pathways (46). Macrophages and other immune cells within the TME can exhibit phenotypic plasticity in response to signals, resulting in dynamic spatiotemporal patterns that influence the immune status and tumor development of the TME. Intensive studies of the complex crosstalk between macrophages and different immune cells have led to a deepening understanding of macrophage-tumor interactions (47).

In the tumor microenvironment, type I helper T (Th1) cells, NK cells, and cytotoxic T lymphocytes (CTLs) can stimulate macrophage polarization toward the M1 type by secreting IFN-γ (48). Polarized M1-type macrophages can release a variety of pro-inflammatory cytokines (TNF-α, IL-6, IL-12, and IL-23) and reactive oxygen/nitrogen intermediates to achieve their tumorigenic activity, and M1-type macrophages can produce chemokines (CXCL9 and CXCL10) to recruit more Th1 cells, thereby creating positive feedback and further amplifying the type I immune response (49). Therefore, M1-type macrophage-mediated immune responses can enhance the antigen-presenting ability of TAMs and effectively improve their antitumor effects.

Interactions between M2-type macrophages and other immune cells (Th2 cells, basophils, regulatory T cells) allow for an enhanced type 2 immune response and contribute to the transformation of tumor cells to malignancy (50). The latter immune cell population induces macrophage polarization towards the M2 type by producing IL-4, IL-13, or IL-10, thus recruiting more Th2 cells into the TME in response to chemokines (CCL17, CCL22, and CCL24) released from activated M2 type macrophages (51). On the other hand, Treg has been shown to promote immunosuppressive responses in macrophages by activating their programmed cell death ligand 1 (PD-L1) (52). Studies have also shown that macrophage function and diversity in TME are also influenced by tumor-infiltrating B cells. Through the production of IL-10 or immunoglobulins, B cells are able to polarize the macrophage population towards the M2 type (53). Tumor-infiltrating M2-type macrophages then inhibit the antigen-presenting ability of dendritic cells (DCs) by producing IL-10 and prevent DCs from activating CTLs, thereby causing dysfunction of DCs in the TME (54). This leads to immune escape and reduces the response of CD8+ T cells to cancer cells. Although the association between macrophages and neutrophils has rarely been reported, new evidence suggests that macrophage depletion in TME can induce the production of highly immunosuppressive neutrophils, the signaling mechanisms of which are currently unknown (55). Overall, macrophages may serve as a global target to regulate innate and adaptive immunity in the TME immune system.

## 4 TAMs and tumors

In early-stage tumors, M1-type macrophages play an anti-tumor immune role and inhibit tumor growth together with T cells and interferon. However, with tumor progression, macrophages gradually lose their tumor-suppressive function and exhibit M2-type tumor-promoting features (56). The role of TAMs in promoting tumor progression is multifaceted. First, TAMs are closely associated with immunosuppressive TME, which is an important cause of the poor prognosis of many human cancers (57). The main manifestation of TAMs immunosuppression is that a higher proportion of M2-type TAMs in the TME leads to increased cancer invasiveness and exacerbates the tumor by generating an immunosuppressive TME, which promotes tumor invasion, metastasis, and progression (58). Second, M2-type TAMs promote angiogenesis; M2-type TAMs are a major source of epidermal growth factor (EGF), which is a direct promoter of tumor growth. Polarized M2-type TAMs constitute a complex cell population including pro-angiogenic macrophages, immunosuppressive macrophages, perivascular macrophages, metastasis-associated macrophages, and invasive macrophages (59). Pro-angiogenic macrophages of TAMs are known to promote tumor growth through the secretion of vascular endothelial growth factor (VEGF), which is an essential component of tumor growth. VEGFA, TGF-β, and angiogenic chemokines (CXCL8 and CXCL12), which promote the activation and recruitment of endothelial cells and fibroblasts in TME. Thus, pro-angiogenic macrophages facilitate tumor angiogenesis and provide sufficient nutrients and oxygen for rapid tumor growth (60). In addition, matrix metalloproteinases (MMPs) and cathepsins produced by M2-type macrophages are able to degrade the surrounding extracellular matrix, which facilitates the migration of cancer cells from the primary tumor tissue. With the expression of angiopoietin 1 receptor, perivascular macrophages can help cancer cells to enter the blood vessels (61). Metastasis-associated macrophages (MAMs) are capable of producing VEGF receptor 1 (VEGFR1), chemokine receptors CXCR3 and CCR2, which provide protection for metastatic cancer cells from removal in the circulatory system (62). In addition to this, there is a strong crosstalk between metastatic cancer cells and MAMs in metastatic tumors. MAMs contribute to the survival of cancer cells, which in turn favors the retention of MAMs in metastatic tumors. It is these important roles of macrophages in tumorigenesis and progression that make them important targets for targeted antitumor therapy.

## 5 Immunotherapeutic strategies targeting TAMs

**Figure 3:**
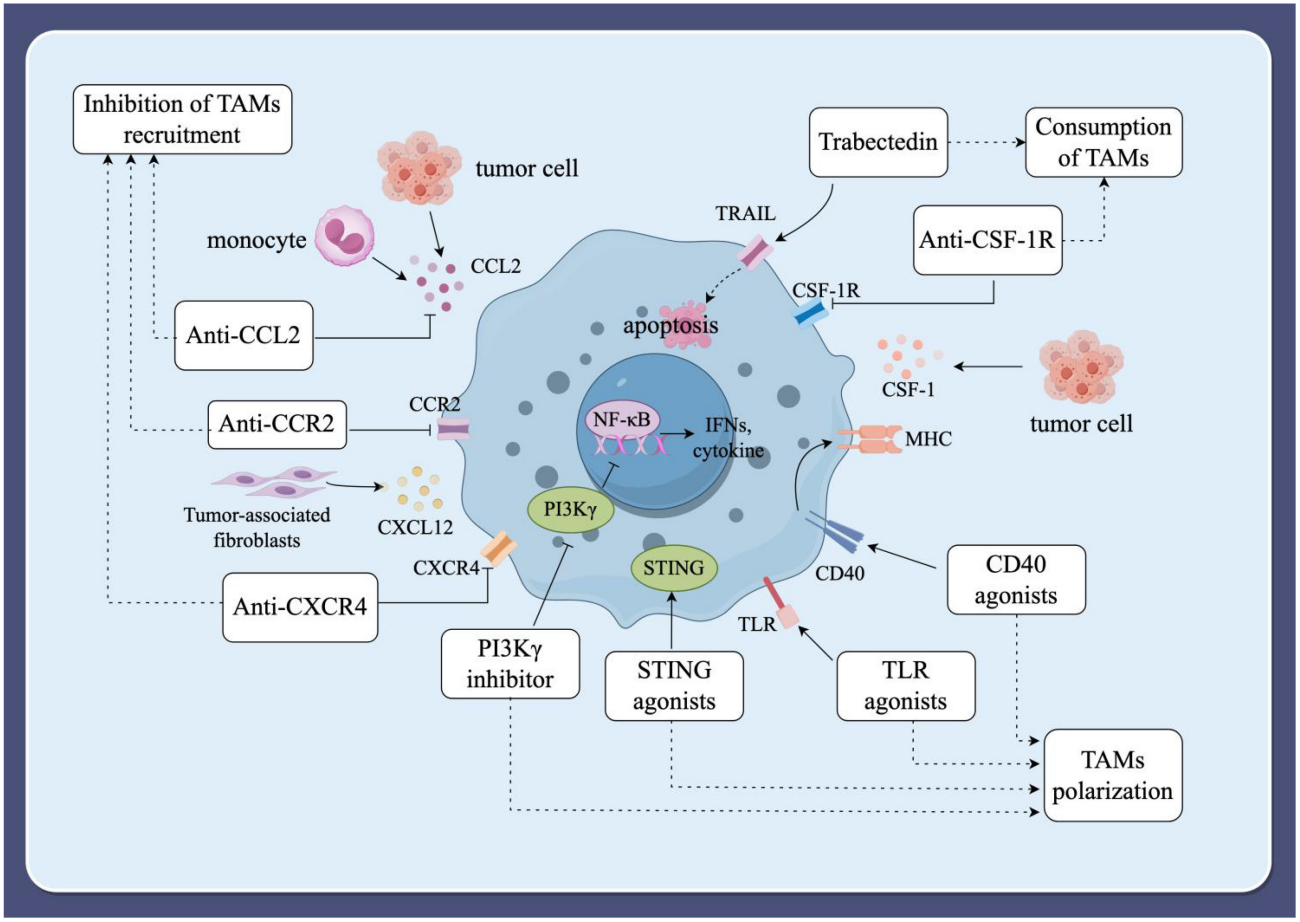
Immunotherapeutic strategies targeting TAMs. (1) Inhibition of TAMs recruitment. The recruitment of peripheral blood mononuclear cells into the TME is facilitated by various tumor-derived chemokines and cytokines, including CCL2, CCL3, CCL4, CCL5, CXCL12, colony-stimulating factor 1 (CSF-1), and VEGF. Inhibiting these factors can block the recruitment of TAMs, thereby slowing tumor progression and metastasis. (2) Consumption of TAMs. Inducing apoptosis in TAMs can also prevent tumor progression. The blockade of the CSF-1/CSF-1R signaling axis and the use of the compound trabectedin are effective strategies to deplete macrophages by inducing apoptosis. (3) Modulating the polarization of TAMs. Modulating macrophage polarization towards the M1-type is an alternative strategy for tumor immunotherapy. Current strategies under investigation include macrophage-targeting antibodies, Toll-like receptor (TLR) agonists, phosphatidylinositol 3-kinase-γ (PI3Kγ) inhibitors, specific nanoparticles, and interferon gene-stimulating factor (STING) agonists.

Macrophages have a dual effect on cancer cells, and their role is multifaceted, allowing for the construction of cancer therapeutic strategies targeting TAMs through multiple pathways (63). Inhibiting the recruitment of TAMs, depleting TAMs, and modulating the polarization of TAMs are all effective ways for cancer therapy (**Figure 3**).

### 5.1 Inhibition of TAMs recruitment

It has been shown that the recruitment of peripheral blood mononuclear cells into TME is achieved by a variety of chemokines and cytokines of tumor origin. These factors include CCL2, CCL3, CCL4, CCL5, and CXCL12, as well as colony-stimulating factor 1 (CSF-1) and VEGF. CCL2 is released by monocytes, tumor cells, and stromal cells in the TME, and its receptor, CCR2, plays an important role in the recruitment of bone marrow-derived monocytes into solid tumors and their development into TAMs. In breast cancer, specific monoclonal antibodies can inhibit the recruitment of TAMs by inhibiting CCL2, thereby delaying tumor progression and metastasis (64). In addition, studies on mouse ovarian cancer models have shown that the anti-tumor effects of anti-CCL2 antibody therapy can be enhanced by combining it with chemotherapy or immunotherapy (65). In conclusion, blocking the CCL2/CCR2 axis is an effective method to inhibit macrophage recruitment in animal models.

CXCL12, a chemokine, induces the transformation of monocytes into M2 macrophages, thereby reducing macrophage activation of T lymphocytes and enhancing macrophage migration, accumulation, and survival in tumors (66). CXCL12 from tumor-associated fibroblasts was found to be able to recruit M2-type macrophages and block CXCR4, the receptor for CXCL12, significantly reducing M2-type macrophage chemotaxis (67). Therefore, disruption of the CXCL12/CXCR4 axis may also be a strategy to inhibit recruitment of TAMs. A study showed that inhibition of the CXCL12/CXCR4 axis suppressed the accumulation of TAMs and sepsis-induced tumor progression in mice (68). However, CXCL12/CXCR4 axis inhibitors have not been reported in human cancer studies. Studies have shown that the CX3CL1/CX3CR1 axis promotes skin carcinogenesis through increased recruitment of M2-type macrophages (69). CX3CL1 is able to promote tumor growth and metastasis in TME (70). Therefore, the CX3CL1/CX3CR1 axis may be a potential target for inhibiting macrophage recruitment, which offers new possibilities for cancer immunotherapy targeting TAMs.

### 5.2 Consumption of TAMs

Induction of apoptosis in TAMs also prevents tumor progression (71). CSF-1, a major growth and differentiation factor released by cancer cells, interacts with its cognate receptor, CSF-1R, which is widely expressed by macrophages and monocytes (72). Blockade of the CSF-1/CSF-1R signaling axis reduces the abundance of macrophages and increases the abundance of CD8+/CD4+ T cells in the TME (73). Studies have shown that high expression of CSF-1 or CSF-1R is associated with poor prognosis in some malignant tumors, such as Hodgkin’s lymphoma and hepatocellular carcinoma (74, 75). Blocking the CSF-1/CSF-1R signaling axis can convert TAMs from a tumor-promoting phenotype to a tumor-killing phenotype (76). Thus, blocking the CSF-1/CSF-1R signaling axis has emerged as a potential strategy for cancer immunotherapy. CSF-1R is a member of the tyrosine kinase receptor family that triggers its own homodimerization and activates receptor signaling upon binding to its ligands, CSF-1 or IL-34 (77). In particular, PLX3397 (pexidartinib), an orally available CSF-1R tyrosine kinase inhibitor, is the most used molecule in clinical studies (78). A study showed a significant reduction in macrophages and delayed tumor growth in mice with mammary tumors after treatment with PLX3397 (79). Tendon-synovial giant cell tumor (TGCT) has become a popular model for studying the CSF-1/CSF-1R signaling axis due to its high expression of CSF-1 and high infiltration of CSF-1R macrophages. A phase 3 trial of TGCT in 2019 demonstrated improved symptoms and prognosis in patients treated with PLX3397, the first drug to show a strong therapeutic effect in TGCT (80). PLX3397, in combination with binimetinib for advanced gastrointestinal mesenchymal stromal tumors and with paclitaxel for advanced ovarian cancer, showed good tolerability and clinical efficacy (81, 82). It’s important to note that this drug includes a boxed warning about the risk of serious and potentially fatal liver injury (83). PLX3397 has also been actively tested in other indications, including melanoma, prostate cancer, and lung cancer, among others. Unfortunately, multiple trials using PLX3397 either alone or in combination with other treatments have been terminated or withdrawn for reasons such as business decisions or insufficient clinical outcomes (NCT02452424, 01499043, 01349036, 01826448, 01090570). One previous clinical trial failed to show efficacy in glioblastoma, despite the fact that adequate drug exposure in tumors had been confirmed. A hypothesis has been proposed that the relative proportion of glioblastoma subtypes might result in treatment resistance; however, correlative studies are still needed to demonstrate this mechanism of resistance (84). This analysis suggests that targeting the CSF-1/CSF-1R signaling axis is a promising strategy for cancer treatment, and CSF-1R inhibitors have great potential to improve the prognosis of patients with advanced cancer.

In addition, some compounds such as trabectedin effectively deplete macrophages by inducing apoptosis. Trabectedin is a second-line antitumor agent that triggers apoptosis in tumor cells by binding to their DNA, resulting in cell cycle arrest and double-stranded DNA breaks (85). Germano et al. (86) found that trabectedin can induce apoptosis of TAMs via the receptor for TNF-related apoptosis-inducing ligand (TRAIL), thereby selectively depleting monocytes or macrophages in both the blood and tumors. Monocytes are highly sensitive to trabectedin-mediated apoptosis due to the low expression levels of TRAIL receptors. In preclinical models, trabectedin has been reported to inhibit the growth and invasion of cutaneous melanoma *in vitro* (87).

Although depletion of TAMs has considerable efficacy in inhibiting tumor progression, precise control of the level and duration of TAM depletion is crucial. Unselective systemic depletion of the entire macrophage population may promote tumor progression (88). Excessive macrophage depletion can disrupt immune homeostasis and increase the risk of infections and autoimmune diseases. Additionally, higher drug doses may be required for long-term TAM depletion, which can lead to adverse effects (89). Therefore, further clinical practice is needed to refine and mature this therapeutic strategy.

### 5.3 Modulating the polarization of TAMs

It is well established that a key feature of TAMs is their plasticity. Modulating macrophage polarization towards the M1-type is an alternative strategy for tumor immunotherapy (90). Current strategies under investigation include macrophage-targeting antibodies, Toll-like receptor (TLR) agonists, phosphatidylinositol 3-kinase-γ (PI3Kγ) inhibitors, specific nanoparticles, and interferon gene-stimulating factor (STING) agonists. Additionally, reprogramming macrophages through genetic engineering techniques, such as the CRISPR-Cas9 genome editing system, offers a promising approach to modulate macrophage polarization.

CD40 is a member of the TNF receptor superfamily expressed on the surface of macrophages. The interaction between CD40 and CD40L initiates the production of pro-inflammatory cytokines and the overexpression of MHC molecules by macrophages. As a result, the tumor-killing function of TAMs can be activated using agonistic anti-CD40 antibodies, thereby restoring their immunosurveillance against tumors (91). A recent study found that the combination of anti-CD40 antibody and anti-IL-6 antibody for glioblastoma reversed TAMs to a tumor-killing phenotype, more effectively inhibiting tumor progression (92). Macrophage receptor with collagenous structure (MARCO) is a scavenger receptor overexpressed on the surface of M2-type TAMs, making it a potential target for cancer therapy (93). Anti-MARCO antibodies can block the inhibitory Fc receptor and reprogram TAMs to the M1-type, thereby inhibiting tumor progression and metastasis.

Macrophages, a major component of the innate immune system, can be activated by pattern recognition receptors and polarized toward the M1 phenotype. Therefore, TLRs agonists can induce macrophage production in the M1 phenotype with potential antitumor effects. In a melanoma tumor model, TLR2 agonists specifically stimulate macrophage polarization toward the M1 phenotype (94). Riquimod (R848), a dual agonist of TLR7 and TLR8, is also able to induce macrophage polarization towards the M1 phenotype. Weissleder and coworkers conducted a large-scale screening assay and designed R848-conjugated cyclodextrin nanoparticles (CDNPs) (95). The R848 Due to the unique advantages of cyclodextrins, CDNPs have a high affinity for TAMs and drug binding affinity, and monotherapy with CDNPs can effectively reduce tumor size and significantly improve survival in mice by modulating the phenotype of TAMs. In 2021, Figueiredo et al. found that the use of lignin nanoparticles (LNPs) conjugated with R848 could reprogram M2 type macrophages to M1 type for enhanced chemotherapy (96). In addition, polyinosinic acid-polycytidylic acid [poly(I:C)], a TLR3 agonist, has also been widely used in cancer therapy due to its potential to activate the immune system (97). In 2020, Dacoba et al. investigated hyaluronic poly(I:C) nanocomplexes, which were shown to be effective at polarizing macrophages to the M1 type with good stability (98).

A number of metabolism-related signaling pathways are important for the altered macrophage phenotype. PI3Kγ controls the expression of arginase 1 (Arg1) and plays a central role in regulating arginine metabolism in immunosuppressed TAMs. Also, pro-inflammatory signaling pathways regulated by nuclear factor kappa-B (NF-κB) activation in macrophages are inhibited by the PI3Kγ pathway (99). Thus, during inflammation and cancer, PI3Kγ controls the switch between immune activation and immune suppression in macrophages. IPI-549 is a specific PI3Kγ inhibitor that downregulates the expression of Arg1, stimulates the activation of NF-κB, and ultimately polarizes macrophages towards the M1-type. An IPI-549 containing polymeric nanoparticles (IPI-549NP) increased the accumulation of IPI-549 at the tumor site and enhanced the anti-tumor immune response (100). In mouse models of pancreatic cancer and melanoma, IPI-549NP promotes an immunostimulatory transcriptional program that activates CD8+ T cells to exert their cytotoxic function and prevents tumor progression by prolonging host survival. In addition, checkpoint inhibitor therapy also benefited from the inhibition of PI3Kγ, as demonstrated by significant tumor regression and enhanced mouse survival in tumor-bearing mice (101). Therefore, activation of anti-tumor immune responses by inhibiting PI3Kγ to polarize macrophages toward M1-type would be a promising therapeutic approach.

Some nanomaterials have a direct impact on immunomodulation by interacting with macrophages (102). Adriamycin, an antitumor drug composed of magnetic iron oxide nanoparticles (IONPs), reprograms TAMs to enable macrophages to exert antitumor effects, which may be useful in enhancing cancer immunotherapy mediated by macrophages (103). A study found that iron-chelated melanin-like nanoparticles could repolarize tumor-promoting M2-type macrophages to M1-type, which could be developed into specialized antigen-presenting cells (APCs) to present tumor-associated antigens induced by photothermal therapy (104). Thus, iron-chelated melanin-like nanoparticles could activate adaptive immune responses and inhibit tumor progression. In a recent study, mannose-chelated iron oxide nanoparticles (man-IONPs) were designed to reprogram TAMs into M1-type macrophages, which had a dramatic inhibitory effect on hepatocellular carcinoma progression (105). In addition, Chen et al. (106) developed an immunotherapeutic gel consisting of anti-CD47 antibody coupled with calcium carbonate nanoparticles. The nanoparticles induced the polarization of TAMs to M1-type, thereby promoting antigen presentation by macrophages to initiate T cell-mediated adaptive immune responses. At the same time, the released anti-CD47 antibody promoted phagocytosis of cancer cells by macrophages.

STING is a cytoplasmic DNA sensor present in a variety of immune cells that controls the transcription of host defense-related genes. When activated by agonists, STING stimulates signaling pathways that cause immune cells to produce a variety of pro-inflammatory cytokines and chemokines, especially type I IFNs that can promote Th1-mediated immune responses (107), and thus STING is able to polarize TAMs to M1 type. However, the route of administration of STING agonists is limited to intra-lesional injections due to their sensitivity to enzymatic degradation, which remains a barrier to successful clinical translation (108). Drug delivery systems developed from nanomaterials can overcome this obstacle. Shae et al. (109) synthesized STING-activated polymeric nanoparticles for the protection of cGAMP delivery, which could transform the tumor immune microenvironment from immunosuppressive to immunogenic and tumor-killing activity. In tumors treated with STING-activated nanoparticles, the percentage of macrophages polarized to M2 type was significantly reduced. In addition, manganese ion (Mn2+) based nano-assemblies were shown to be a STING agonist that promotes anti-tumor therapy by initiating the immune system (110). In different tumor models, significant therapeutic effects were demonstrated using very small doses of STING agonists and the population of TAMs showed an increase in the M1/M2 ratio, suggesting a conversion of TAMs to the M1 type (111).

The CRISPR-Cas9 genome editing system has great potential in cancer therapy due to its ability to precisely target key oncogenes and tumor suppressors (112). Current clinical trials using CRISPR-Cas9 for cancer therapy have focused on isolating and extracting T cells from patients, subjecting them to CRISPR-Cas9-mediated gene editing, and subsequently re-injecting them into patients. However, the safe and effective manipulation of specific genomic sequences in the tumor microenvironment remains a major challenge for the clinical application of CRISPR-Cas9 in cancer therapy. The presence of M1-type TAMs correlates with antitumor activity, whereas the presence of M2-type TAMs correlates with pro-tumor activity. Using CRISPR-Cas9, several relevant genes can be knocked out to permanently reprogram TAMs into an anti-tumor M1-like phenotype while maintaining their adaptive properties. These reprogrammed macrophages can sustain their anti-tumor effects without succumbing to the immunosuppressive tumor microenvironment, thus maximizing the efficacy of gene editing therapy. Therefore, TAMs are also important targets for enhancing the efficacy of gene editing in cancer treatment. A recent study developed an *in vivo* CRISPR-Cas9 targeting system for TAMs using bacterial protoplast-derived nanovesicles (NVs) (113). In this system, plasmid-transformed E. coli protoplasts were used as a production platform, and the vesicles were modified with pH-responsive PEG-conjugated phospholipid derivatives and galactosamine-conjugated phospholipid derivatives tailored for TAM targeting. These vesicles were loaded with DNA fragments targeting the macrophage-polarized Cas9-sgRNA ribonucleoprotein, Pik3cg, and the ligand for TLR9, CpG. The bacteriophage-derived exosomes, loaded with CRISPR-Cas9 tools, remodeled the tumor microenvironment by stabilizing the M1-like phenotype in TAMs, thereby inhibiting tumor growth in female mice. These findings pave the way for cancer immunotherapy by overcoming challenges associated with maintaining the activity, safety, and precisely targeted delivery of gene-edited cells *in vivo*.

## 6 Conclusion and future perspectives

In recent years, research on the diagnosis and treatment of macrophage-associated diseases, especially cancer, has made remarkable progress (114). In the tumor microenvironment, TAMs mainly exhibit M2-type tumor-promoting features, and the abundant presence of TAMs is closely related to tumor recurrence and metastasis (115). By inhibiting the recruitment of TAMs, depleting TAMs, and modulating the polarization of TAMs, targeted TAM therapy has made great progress. However, there are still many issues that need to be further studied and explored. The mechanism of macrophage differentiation and diversity in different tissues is still an important issue that remains to be resolved, and the various functional characteristics of macrophages in TME are closely related to macrophage differentiation and diversity. Currently, the assessment of heterogeneous macrophages is usually limited to the macrophage population, and elucidating macrophage heterogeneity at the single-cell level remains a great challenge. More fundamental studies of macrophage phenotype and function, and thus elucidation of the dual effects of macrophages on tumors, could inform more specific therapeutic strategies for targeting TAMs (116).

Despite the tremendous success of TAMs-targeting strategies against tumors, TAMs continue to contribute to chemoresistance in a variety of cancers due to the complexity of macrophage effects on tumors. Important mechanisms include M2 macrophage-induced epithelial mesenchymal transition, M2 macrophage production of metabolites, and M2 macrophage-induced production of anti-apoptotic signals (117, 118). The stimulatory effects produced by M2 macrophages can severely affect the efficacy of clinical radiotherapy. Therefore, targeting TAMs as a complementary therapy, in synergy with radiotherapy, chemotherapy, or immunotherapy, may help counteract drug resistance in cancer treatment to some extent.

In conclusion, despite their negative role in tumor development, tumor-associated macrophages have great potential in tumor therapy due to their critical role as an important component of the tumor microenvironment. Targeting macrophages or integrating them with radiotherapy, chemotherapy, and immune checkpoint inhibitors has a significant impact on tumor therapy. Specifically, eliminating tumor-promoting macrophages while simultaneously administering antitumor drugs significantly improves tumor killing. Moreover, targeting pathways both upstream and downstream of macrophages offers additional therapeutic avenues to modulate macrophage function. Notably, the use of genetic engineering to reprogram macrophages to convert tumor-promoting macrophages into antitumor macrophages presents a highly promising clinical application. With a deeper understanding of tumor-associated macrophages in the future, it is anticipated that this knowledge will provide a useful reference for designing more precise treatment plans and potentially lead to new breakthroughs in the field of tumor therapy.
